# Copper Chaperone CupA and Zinc Control CopY Regulation of the Pneumococcal *cop* Operon

**DOI:** 10.1128/mSphere.00372-17

**Published:** 2017-10-18

**Authors:** Miranda J. Neubert, Elizabeth A. Dahlmann, Andrew Ambrose, Michael D. L. Johnson

**Affiliations:** a Department of Immunobiology, University of Arizona, Tucson, Arizona, USA; b Department of Pharmaceutical Sciences, University of Arizona, Tucson, Arizona, USA; c Bio5 Institute, University of Arizona, Tucson, Arizona, USA; Martin Luther University of Halle-Wittenberg

**Keywords:** chaperones, copper, heavy metals, metal, metal resistance, operon, pneumococcus, repressor, *Streptococcus pneumoniae*, zinc

## Abstract

Any metal in excess can be toxic; therefore, metal homeostasis is critical to bacterial survival. Bacteria have developed specialized metal import and export systems for this purpose. For broadly toxic metals such as copper, bacteria have evolved only export systems. The copper export system (*cop* operon) usually consists of the operon repressor, the copper chaperone, and the copper exporter. In *Streptococcus pneumoniae*, the causative agent of pneumonia, otitis media, sepsis, and meningitis, little is known about operon regulation. This is partly due to the *S. pneumoniae* repressor, CopY, and copper chaperone, CupA, sharing limited homology to proteins of putative related function and confirmed established systems. In this study, we examined CopY metal crosstalk, CopY interactions with CupA, and how CupA can control the oxidation state of copper. We found that CopY bound zinc and increased the DNA-binding affinity of CopY by roughly an order of magnitude over that of the apo form of CopY. Once copper displaced zinc in CopY, resulting in operon activation, CupA chelated copper from CopY. After copper was acquired from CopY or other sources, if needed, CupA facilitated the reduction of Cu^2+^ to Cu^1+^, which is the exported copper state. Taken together, these data show novel mechanisms for copper processing in *S. pneumoniae*.

**IMPORTANCE** As mechanisms of copper toxicity are emerging, bacterial processing of intracellular copper, specifically inside *Streptococcus pneumoniae*, remains unclear. In this study, we investigated two proteins encoded by the copper export operon: the repressor, CopY, and the copper chaperone, CupA. Zinc suppressed transcription of the copper export operon by increasing the affinity of CopY for DNA. Furthermore, CupA was able to chelate copper from CopY not bound to DNA and reduce it from Cu^2+^ to Cu^1+^. This reduced copper state is essential for bacterial copper export via CopA. In view of the fact that innate immune cells use copper to kill pathogenic bacteria, understanding the mechanisms of copper export could expose new small-molecule therapeutic targets that could work synergistically with copper against pathogenic bacteria.

## Abstract (precis)

As mechanisms of copper toxicity are emerging, bacterial processing of intracellular copper, specifically inside *Streptococcus pneumoniae*, remains unclear. In this study, we investigated two proteins encoded by the copper export operon: the repressor, CopY, and the copper chaperone, CupA. Zinc suppressed transcription of the copper export operon by increasing the affinity of CopY for DNA. Furthermore, CupA was able to chelate copper from CopY not bound to DNA and reduce it from Cu^2+^ to Cu^1+^. This reduced copper state is essential for bacterial copper export via CopA. In view of the fact that innate immune cells use copper to kill pathogenic bacteria, understanding the mechanisms of copper export could expose new small-molecule therapeutic targets that could work synergistically with copper against pathogenic bacteria.

## INTRODUCTION

Metals are necessary components for maintaining protein structure, stability, and function. Intracellular metal levels are controlled by highly regulated acquisition and efflux mechanisms. These efflux systems are required to decrease internal metal toxicity that arises from metal intoxication. Copper is broadly toxic to many organisms, especially bacteria (1–6). Copper can disrupt multiple bacterial cellular processes, including iron-sulfur clusters, glutamate synthesis, and deoxynucleoside triphosphate synthesis (7–9). The immune system takes advantage of this toxicity by importing copper into an oxidized phagolysosome to kill pathogens (8, 10). This antimicrobial effect has been exploited since antiquity and is currently used in hospitals to reduce nosocomial infections (11, 12). Several different types of bacterial copper export systems are used to mitigate toxic effects. In general, these systems consist of an operon repressor that releases DNA upon copper binding, a copper chaperone, and the exporter. Further understanding of how copper export systems are regulated in different bacterial pathogens is an important step in combating these infections.

*Streptococcus pneumoniae* (the pneumococcus) is a Gram-positive pathogen and a causative agent of pneumonia, otitis media, and meningitis. Often residing in the upper respiratory tract, the pneumococcus encounters innate immune responses that use copper to combat infection (3). The pneumococcus contains a copper export system, the *cop* operon, that encodes a repressor, CopY, the copper chaperone, CupA, and the copper exporter, CopA. CopA, which exports Cu^1+^ from the bacterium, is important for pneumococcal virulence and resistance to copper toxicity (3, 13–15).

*S. pneumoniae* CopY is highly conserved (>90% identity) in many streptococcal species and is related to other copper operon repressors, such as COPR in *Lactococcus lactis* (34% identity, 57% similarity) (16). *S. pneumoniae* CopY protein structure predictions reveal similarity to the *L. lactis* COPR winged helix-turn-helix DNA-binding domain (see Fig. S1A and C in the supplemental material) (17, 18). The *L. lactis* COPR nuclear magnetic resonance structure (PDB code 2K4B) contains only the sequence of the DNA-binding domain and therefore does not include the metal-binding domain. Thus, how CopY interacts with copper or other metals remains unknown. The known protein structure most homologous to the generated pneumococcal CopY model is that of BlaI (PDB code 1SD4), the penicillinase repressor in *Staphylococcus aureus* (Fig. S1B and D) (19). There are several structures of copper operon repressors/sensors containing copper, such as in CueR from *Escherichia coli* and CsoR from *Mycobacterium tuberculosis* (16, 20, 21). However, these proteins share no homology with pneumococcal CopY.

10.1128/mSphere.00372-17.2 FIG S1  Alignment homology modeling of *S. pneumoniae* CopY. (A, B) *L. lactis* COPR (A) aligned with *S. pneumoniae* CopY and *S. aureus* BlaI (B) aligned with *S. pneumoniae* CopY. In each case, identity is assessed by using identical residues but not similar residues, which is indicated by a plus sign. The E (expect) value is a measure of the number of sequence hits that would be expected and is inversely correlated with the number of matching or similar residues. (C, D) *L. lactis* COPR DNA-binding domain (C) (PDB code 2K4B) and *S. aureus* Bla (D)I (PDB code 1SD4) aligned with an I-TASSER prediction of *S. pneumoniae* CopY by using PyMOL. Download FIG S1, TIF file, 0.7 MB. Copyright © 2017 Neubert et al. 2017 Neubert et al. This content is distributed under the terms of the Creative Commons Attribution 4.0 International license.

A further feature distinguishing pneumococcal CopY from other homologous proteins is the lack of a C-terminal CXC Cu^1+^-binding motif compared with COPR from *L. lactis*, while the beta-lactamase repressor from *S. aureus* lacks both CXC motifs (Fig. S1A). The number of CXC motifs could affect the coordination and number of copper or other metal atoms in CopY. Interestingly, Zn^2+^ can be displaced by two Cu^1+^ atoms in *Enterococcus hirae* CopY (22). Additionally, zinc was shown to suppress copper-induced pneumococcal *cop* operon expression while bacterial zinc export is upregulated upon copper stress (8, 13). The mechanism of how zinc, copper, and other metals interact with COPR/CopY repressors in context with DNA binding remains unclear.

In contrast to CopY, the *S. pneumoniae* CupA sequence is unique and is present in only a few streptococcal species. Although the CupA copper chaperone is not needed for virulence in mice, it is required for copper resistance in culture (3, 13, 23). Copper chaperones in different organisms have been shown to traffic Cu^1+^ both to the membrane copper exporter CopA as CupA in *S. pneumoniae* and to the operon repressor COPR as CopZ in *E. hirae* (22, 23). Notably, *E. hirae* CopZ and pneumococcal CupA share no homology. *S. pneumoniae* CupA differs from other copper chaperones in that it has a cupredoxin fold and, as a result, may play a role in reducing or facilitating the reduction of copper inside the bacteria for efficient export by CopA. Such activity would also facilitate the need for the copper chaperone to also coordinate Cu^2+^.

In this study, we examined the influence of copper and other metals on CopY, CupA’s effect on copper, and the interplay between the two proteins. We tested how *S. pneumoniae* CopY interacts with copper to release it from the *cop* operon and how this process is affected by zinc, if CupA could facilitate the copper reduction, and/or if CupA could affect CopY’s repressor function. We found that zinc caused tetramerization of CopY and thus increased its affinity for DNA by approximately an order of magnitude, CupA facilitated the reduction of Cu^2+^ to Cu^1+^, and CupA chelated copper from CopY.

## RESULTS

### CopY binds copper to upregulate the *cop* operon.

**FIG 1:**
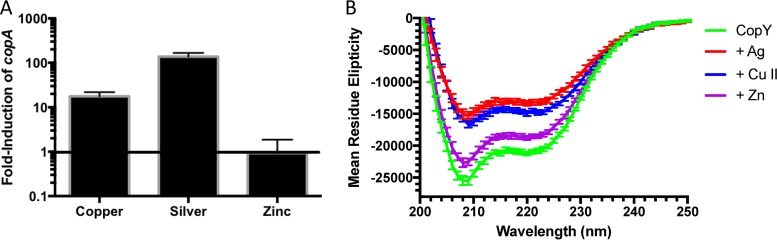
CopY repression of *cop* operon expression is controlled by metal stress. (A) Real-time PCR assay of *copA* in response to Cu^2+^, Ag^2+^, or Zn^2^ at 100 µM compared to no metal stimulation. Error bars are standard deviations across three independent experiments. (B) CD mean residue ellipticity of 10 µM apo-CopY or 10 µM CopY with 50 µM Ag, Cu^2+^, or Zn^2+^. Data are plotted as the average of four individual experiments.

Although the well-established model of copper export in *E. coli* involves copper driving expression of the *cop* operon, *copA* expression is also upregulated by CueR in the presence of excess silver but not in that of excess zinc (24, 25). Even though the mechanism of copper efflux system activation is distinct in these two systems, we determined whether these metals affect *S. pneumoniae cop* operon regulation. We measured *copA* expression as a readout of *cop* operon regulation under conditions of metal stress. Consistent with previous reports, silver upregulated *copA* expression more than equimolar amounts of Cu^2+^, while zinc had no effect (Fig. 1A) (13, 26). However, this result could simply be due to silver being more toxic or *S. pneumoniae* being less efficient at exporting silver. Additionally, zinc’s inability to upregulate the *cop* operon is not an indication of CopY’s ability to bind zinc.

To determine whether the ability to derepress the *cop* operon correlated with a change in CopY secondary structure, CopY was examined by circular dichroism (CD) complexed to various metals (Table S1). Both apo CopY and Zn^2+^-bound CopY yielded the highest mean residue ellipticity signal at 208 to 222 nm, which is representative of alpha helicity, compared to copper- and silver-bound CopY (Fig. 1B). Taken together, the data show that the change in the alpha-helical signal of CopY with copper and silver bound provides some evidence of a metal-dependent change in secondary structure. This result is consistent with a conformational change affecting how a protein with a high affinity for DNA could be converted into one with a low affinity for DNA.

10.1128/mSphere.00372-17.1 TABLE S1  Strains used in this study. Download TABLE S1, TIF file, 1 MB. Copyright © 2017 Neubert et al. 2017 Neubert et al. This content is distributed under the terms of the Creative Commons Attribution 4.0 International license.

### CopY changes affinity for the *cop* operon and oligomerization state under different metal conditions.

**FIG 2:**
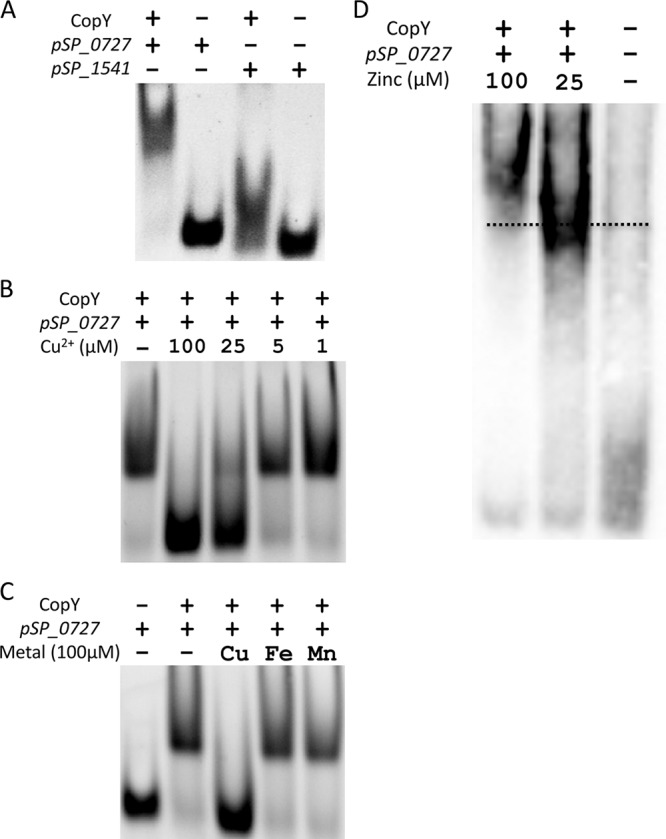
CopY repression of the *cop* operon is controlled by metal stress. (A) EMSA of upstream DNA of the *cop* operon with 50 µM CopY binding to 50 ng of promoter DNA (*pSP_0727*) or negative-control promoter DNA (*pSP_1541*). Data are representative of at least three individual experiments unless otherwise noted. (B) EMSA with 50 ng of *pSP_0727*, 50 µM CopY, and decreasing concentrations of copper. (C) EMSA with 50 ng of *pSP_0727*, 50 µM CopY, and 100 µM Cu^2+^, Fe^3+^, or Mn^2+^. (D) EMSA with 50 ng of *pSP_0727* and 50 µM CopY in the presence of 25 or 100 µM zinc. Gel was run at 100 V for 60 min to emphasize the shift size difference. The dotted line shows the midpoint of CopY-DNA binding with no metal present.

To determine empirically the effects of CopY in the release of DNA in the presence of copper, we performed electrophoretic mobility shift assays (EMSAs) and fluorescence polarization assays. We first needed to identify the site in the pneumococcal genomic DNA where CopY binds. We synthesized a biotinylated fragment of double-stranded DNA starting from −71 to −14 before the *cop* operon (*pSP_0727*), which contains consensus CopY regulatory sequences TACANNTGTA and TATACTNNNNNNTGTAAT (13, 17, 27–29). We also chose an unrelated pneumococcal promoter region upstream of *SP_1541* as a negative control (*pSP_1541*). CopY bound to *pSP_0727* in a protein and DNA concentration-dependent manner (Fig. 2A; Fig. S2A and B). In contrast, no binding to *pSP_1541* was observed (Fig. 2A). Although slightly complicated by EDTA in the EMSA gels, CopY binding to *pSP_0727* was disrupted by the addition of copper and silver but not by the addition of manganese, which has previously been shown to rescue copper toxicity, or iron (Fig. 2B and C; Fig. S2C) (8).

10.1128/mSphere.00372-17.3 FIG S2  CopY binds DNA in a protein-dependent manner. (A) EMSA showing increasing concentrations of CopY with a constant amount of DNA (50 ng of *pSP_0727*). Data are representative of three individual experiments. (B) EMSA with decreasing amounts of *pSP_0727* DNA with a constant amount of CopY protein (starting with 50 µM). Data are representative of three individual experiments. (C) EMSA of upstream DNA of the *cop* operon with 50 µM CopY binding to 50 ng of promoter DNA in the presence 100 µM AgNO_3_. Download FIG S2, TIF file, 2.5 MB. Copyright © 2017 Neubert et al. 2017 Neubert et al. This content is distributed under the terms of the Creative Commons Attribution 4.0 International license.

Although zinc binding did not derepress expression of the *cop* operon or change the secondary structure of CopY to the same degree as copper and silver binding, which can upregulate expression of the *cop* operon, we found that it could induce other changes. For example, copper repressors have previously been shown to bind zinc, which results in multimerization (22, 30). Additionally, zinc export is upregulated under copper stress in the pneumococcus (8). Thus, we determined whether zinc interacts with CopY’s ability to bind DNA and form multimeric structures. In the EMSA, we saw an upward shift occur in a zinc-dependent manner, implying that pneumococcal CopY was multimerizing in the presence of zinc (Fig. 2D). To further investigate the multimerization of CopY in the presence of zinc, we performed size exclusion chromatography with multiangle light scattering (SEC-MALS) to ascertain the molecular mass in both the apo and zinc-bound states. Because of the construct used, the CopY monomer should be 18.1 kDa. However, the apo form of CopY appeared at a molecular mass of 40.9 kDa, implying that without a metal bound, CopY exists as a dimer (Fig. S3A, B, and E). Interestingly, the molecular mass of the zinc-bound form of CopY was 79.4 kDa, suggesting that it exists as a tetramer in solution (Fig. S3C, D, and E). Taken together, the results show that zinc caused the CopY dimer to dimerize into a tetramer, a form consistent with other copper operon repressors (30–32).

10.1128/mSphere.00372-17.4 FIG S3  SEC-MALS of CopY. Data are plotted as a molar mass distribution (red) superimposed on the chromatogram of absorbance at 280 nm as a function of the elution volume. CopY is shown without zinc in panel A and with zinc in panel C. Panels B and D are the same molar mass distribution (blue) graphs with the signal distribution absorbance at 280 nm shown in green, while light scattering (LS) is in black and the differential refractive index (RI) is in red for the apo form and with zinc, respectively. (E) Molar masses determined with theoretical values. Superscripts: a, name of sample (the applied volume is 100 µl in all cases); b, peak number from first to last with the mass of the protein (µg) in the peak in parentheses; c, molar mass (Mp) of protein at the plateau of the peak with a theoretical molecular mass of the monomer protein in kDa in parentheses; d, weight average molar mass determined via MALS; e, polydispersity of mass in peak. Download FIG S3, TIF file, 0.4 MB. Copyright © 2017 Neubert et al. 2017 Neubert et al. This content is distributed under the terms of the Creative Commons Attribution 4.0 International license.

**FIG 3:**
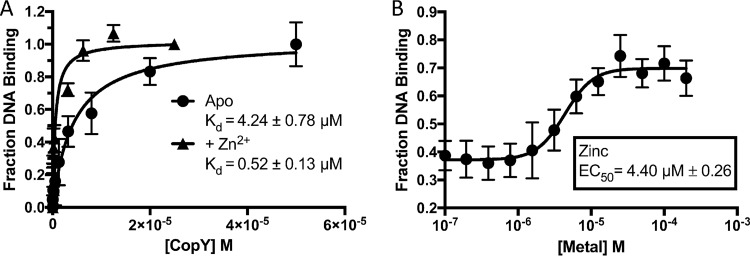
Zinc increases binding of CopY to the *cop* promoter. (A) Fluorescence polarization assay of CopY binding to 10 nM p*SP_0727* with FAM and with and without 10 µM zinc. Curve fit to one-site binding (hyperbola). Data are representative of three individual experiments. (B) Fluorescence polarization assay of CopY binding to 10 nM p*SP_0727* with FAM in the presence of Zn^2+^ starting with the DNA and protein concentrations required for 40% binding in panel A (2 µM CopY and 10 nM DNA). Curve fit to sigmoidal, four-parameter logistic, where *x* is the log concentration. Data are representative of four individual experiments.

Next, we determined the binding affinity of CopY for *pSP_0727* and what effect zinc multimerization has on that binding. Using fluorescence polarization assay, we observed a 4.24 ± 0.78 µM binding affinity for apo CopY to *pSP_0727* and nonspecific binding to the negative control, *pSP_1541* (Fig. 3A; Fig. S4). Binding increased by almost an order of magnitude with the addition of Zn^2+^ to 0.52 ± 0.13 µM (Fig. 3A). Additionally, starting with a protein concentration that led to 40% CopY binding to *pSP_0727*, we observed an increase in signal in the presence of Zn^2+^ with a 50% effective concentration of 4.40 µM (Fig. 3B). Taken together, the results show that CopY had a higher affinity for the *cop* operon promoter in the presence of zinc binding, which could be due to the tetrameric state of CopY.

10.1128/mSphere.00372-17.5 FIG S4  CopY binding is specific to the *cop* operon promoter. Fluorescence polarization (FP) assay of various amounts of CopY binding to 10 nM *cop* promoter (*SP_0727* promoter with FAM) or to 10 nM negative control (*SP_1541* promoter with FAM). Curve fit to one-site binding (hyperbola) or linear line where no substrate-dependent increase in binding occurred. Data are representative of four individual experiments. Download FIG S4, TIF file, 0.02 MB. Copyright © 2017 Neubert et al. 2017 Neubert et al. This content is distributed under the terms of the Creative Commons Attribution 4.0 International license.

### Combinatory effects of copper and zinc in pneumococcus killing.

**FIG 4:**
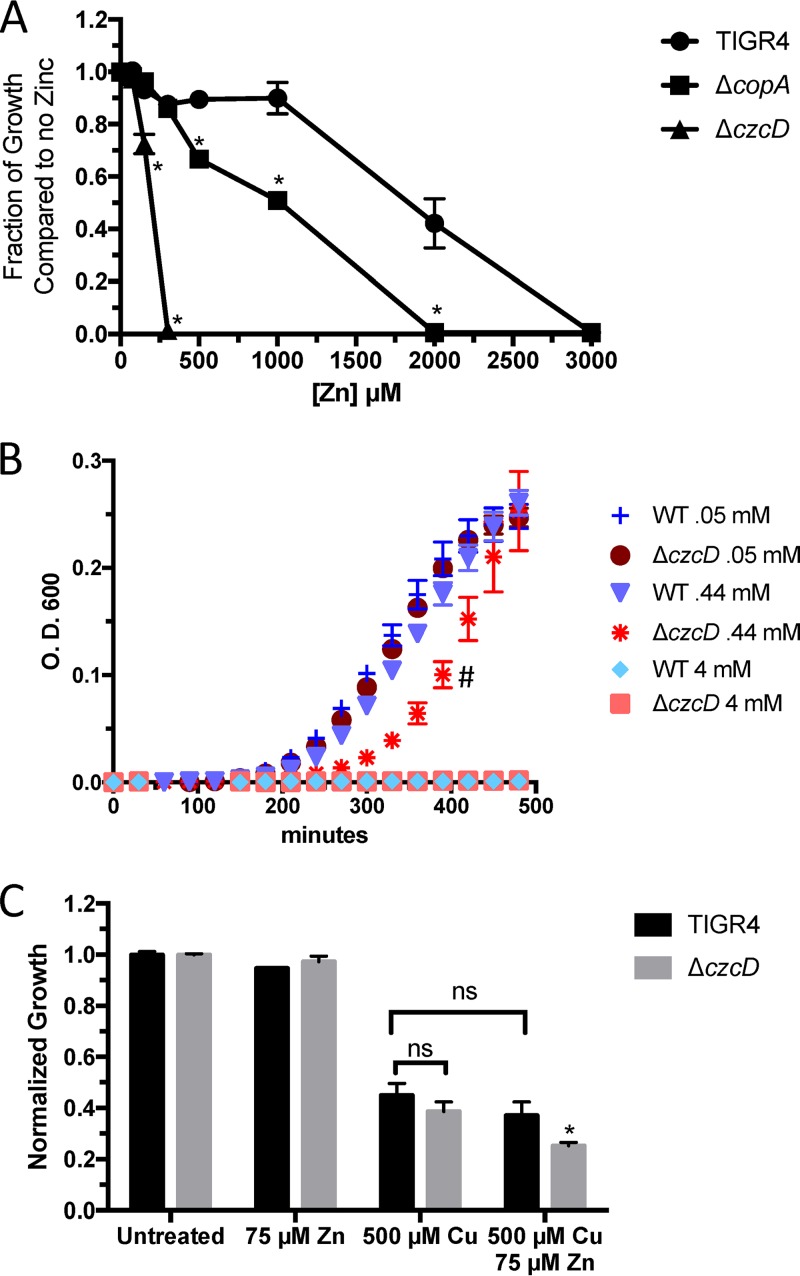
Inability to export zinc increases copper stress. (A) Endpoint growth of wild-type *S. pneumoniae* TIGR4 and Δ*copA*, Δ*czcD*, and Δ*copA ΔczcD* mutants in the presence of various concentrations of Zn^2+^ after 18 h of growth in THYB at pH 6.5 at 37°C. Error bars represent standard deviations and are representative of at least three independent experiments. *, *P* < 0.01 in a Student *t* test. (B) Timed growth curve of wild-type (WT) TIGR4 and the Δ*czcD* mutant under various concentrations of copper stress in THYB at pH 6.5 at 37°C. #, *P* < 0.01 in a paired *t* test. (C) Endpoint growth of wild-type *S. pneumoniae* TIGR4 and the Δ*czcD* mutant in a nonlethal concentration of Zn^2+^ (75 µM) and approximately the 50% lethal dose of Cu^2+^ (500 µM) after 18 h of growth in THYB at pH 6.5 at 37°C. *, *P* < 0.02 in a Student *t* test. ns, no significant difference.

To examine if the increased affinity of CopY for DNA with zinc present has a detrimental effect on bacterial survival under copper stress, we made a Δ*czcD* (*SP_1857* zinc exporter) mutant of wild-type *S. pneumoniae* (TIGR4) to increase the internal concentration of zinc (Table S1) (3, 8). As expected, in an overnight growth assay, the Δ*czcD* mutant was significantly more susceptible to zinc stress than the wild-type and Δ*copA* mutant strains, and the Δ*copA* mutant strain was more susceptible to zinc stress than wild-type *S. pneumoniae* (Fig. 4A). Additionally, there was a significant growth lag of the Δ*czcD* mutant under copper stress compared to *S. pneumoniae* TIGR4 (Fig. 4B). When combined with a sublethal dose of zinc (75 µM), copper was more toxic to the Δ*czcD* mutant than to *S. pneumoniae* TIGR4 (Fig. 4C). Taken together, the results show that excess zinc is detrimental to the ability of the pneumococcus to combat copper stress and sublethal amounts of zinc with copper have additive effects that inhibit bacterial growth.

### CupA facilitates copper reduction.

**FIG 5:**
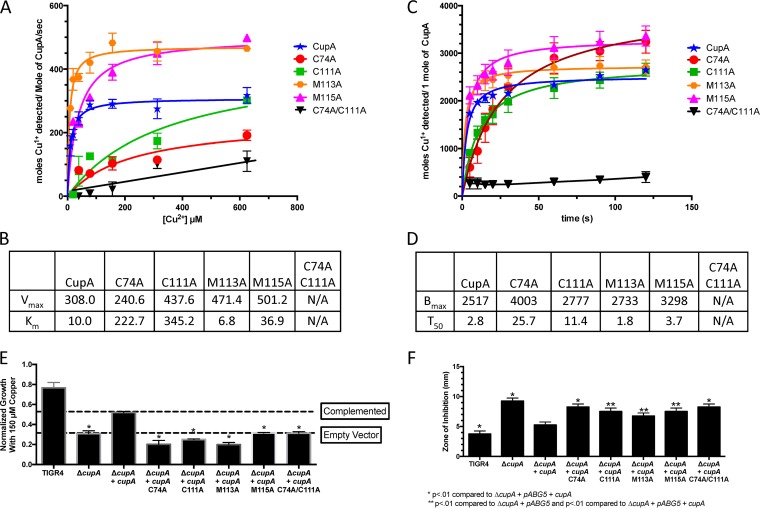
CupA’s ability to reduce Cu^2+^ is necessary to prevent copper stress. (A) Maximum rate of CupA Cu^2+^ reduction and release of Cu^1+^, as determined by BCS, for recombinant CupA and various mutations of the Cu^1+^-binding domain, as described by Fu et al. (14). Each error bar represents the standard error of the mean of three experiments. The rate was averaged over a 5-s interval. (B) *V*_max_ and *K*_*m*_ of wild-type CupA and variants as determined by curve fits to a Michaelis-Menten model. (C) CopY Cu^2+^ reduction and release of Cu^1+^ over time, as determined by BCS, for recombinant CupA and various mutations of the Cu^1+^-binding domain at a 312.5 µM Cu^2+^ concentration. Each error bar represents the standard error of the mean of three experiments. (D) *B*_max_ and *T*_50_ of wild-type and mutant forms of CupA as determined by curve fits to a hyperbola one-site model fit. (E) Endpoint growth of *S. pneumoniae* TIGR4, the Δ*cupA* mutant, the Δ*cupA* mutant with the empty pABG5 vector, and the Δ*cupA* mutant complemented with wild-type and various mutant forms of *cupA* at 150 µM Cu^2+^ in THYB at pH 6.5 at 37°C. *, *P* < 0.01 compared to the Δ*cupA* mutant complemented with wild-type *cupA* on the pABG5 vector in a Student *t* test. Each strain, except TIGR4, contains pABG5. Data are representative of three independent experiments with three replicates. (F) Zone of inhibition of *S. pneumoniae* TIGR4, the Δ*cupA* mutant, the Δ*cupA* mutant with the empty pABG5 vector, and the Δ*cupA* mutant complemented with wild-type and various mutant forms of *cupA* on TSA blood agar plates incubated overnight at 37°C. Zones were measured as the distance from the edge of the disc containing 10 µl of 1 M Cu^2+^. *, *P* < 0.01 compared to the Δ*cupA* mutant complemented with wild-type *cupA* on the pABG5 vector; **, *P* < 0.01 compared to the Δ*cupA* mutant complemented with wild-type *cupA* on the pABG5 vector and the Δ*cupA* mutant with the empty pABG5 vector in a Student *t* test. Data are representative of three independent experiments with six replicates each. N/A, not available.

In previous toxicity assays, copper was added to the pneumococcus as Cu^2+^ under nonreducing conditions (8, 13). Furthermore, it has been shown in *E. coli* that copper can enter the bacterium as Cu^2+^ via the iron siderophore Ybt (33, 34). However, for copper to leave the bacterium, it has to be in the Cu^1+^ redox state and therefore must be reduced for functional CopA export (14, 25). In *S. pneumoniae*, CupA has a predicted cupredoxin fold and a membrane leader sequence, which it uses to shuttle bound Cu^1+^ to the membrane-bound copper exporter CopA (23). Using the Cu^1+^ colorimetric reporter bathocuproinedisulfonic acid (BCS) and the crystal structure of CupA to design amino acid exchanges, we determined whether CupA could facilitate the reduction of Cu^2+^ to Cu^1+^ and which amino acid residues are involved in that reaction (Table S1) (23). Because the leader sequence made CupA insoluble, we used CupA amino acids 23 to 123 for biochemical assays. In these assays, there is incomplete accessibility of Cu^1+^ to BCS when using the CupA protein because the signal depends upon CupA’s ability to reduce and release Cu^1+^. Given these caveats, we still observed a reduction of Cu^2+^ to Cu^1+^ that was not detected with buffer, Cu^2+^, and BCS alone (Fig. 5A to D). Thus, recombinant CupA is able to reduce Cu^2+^ to Cu^1+^ in a manner detectable by a Cu^1+^ chelator.

Next, we wanted to examine the residues involved in this reduction. The CupA structure contains two Cu^1+^ atoms: one coordinated by C74 and C111 (the high-affinity site) and one coordinated by C74, M113, and M115 (the low-affinity site) (23). Therefore, we mutated each of these residues to alanine and also made a double cysteine mutant form to represent a completely null mutant form (Table S1). The C74A C111A double variant was unable to facilitate the reduction of Cu^2+^ to Cu^1+^ in a concentration-dependent manner, suggesting that both of these residues are used to catalyze copper reduction. However, each single cysteine variant was independently able to catalyze the reaction, albeit significantly more slowly than wild-type CupA (Fig. 5A to D). This result implies that C74 and C111 are at least sterically involved in the coordination of Cu^2+^ before reduction takes place. Additionally, a time course at a fixed concentration confirmed that the double amino acid exchange variant was unable to facilitate the reduction of copper (Fig. 5C and D). The M113A and M115A mutants yielded higher rates and *V*_max_ values than did wild-type CupA (Fig. 5A and B). The increases in the M113A and M115A mutants’ *V*_max_ values are consistent with the assay in that BCS needs to detect the CupA reduced and released Cu^1+^ and these exchanges were made to the protein’s low-affinity Cu^1+^-binding domain (23).

### CupA’s role in alleviation of copper toxicity.

To examine the effect of these mutations on resistance to copper toxicity, we put full-length wild-type and full-length point mutant versions of *cupA* in a pABG5 vector and introduced them into the *S. pneumoniae* TIGR4 Δ*cupA* background (Table S1). In both endpoint growth curve and zone-of-inhibition assays, each Cu^1+^-coordinating residue was necessary to restore wild-type *cupA*-complemented copper resistance in the bacteria (Fig. 5E and F). Taken together, these results show that although the single amino acid exchanges still allowed copper reduction, each copper-binding residue was necessary to restore wild-type resistance to copper toxicity.

### CupA’s effect on CopY’s DNA binding.

**FIG 6:**
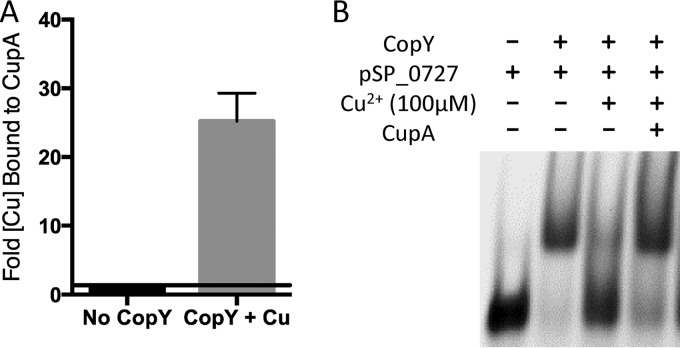
CupA chelates copper from CopY. (A) Inductively coupled plasma mass spectrometry showing the fold increase in the total amount of copper bound to TRV protease-cleaved CupA after chelation of copper from CopY bound to nickel resin. (B) EMSA with 50 ng of *pSP_0727*, 50 µM CopY, 100 µM Cu^2+^, and 100 µM wild-type CupA.

Metallochaperones can provide copper to sensors and exporters (4, 22, 23). CupA has already been shown to take copper to the CopA exporter (23). Therefore, we examined interactions between pneumococcal CopY and CupA. We determined whether CupA could chelate copper from CopY and if this chelation could restore CopY’s ability to bind to DNA in the presence of copper. We found that, *in vitro*, CupA can restore binding of CopY to DNA in the presence of Cu^2+^ by chelating copper from CopY (Fig. 6A and B). These results are consistent with CupA having a membrane leader sequence and thus not trafficking copper to the repressor for activation the operon but instead for the copper to be chelated from copper-bound CopY so that it can return to repress the *cop* operon (23).

## DISCUSSION

Here we have shown that, *in vitro*, zinc can lead to homotetramerization of CopY, which binds upstream of the pneumococcal *cop* operon, in addition to the protein existing as a dimer in the apo form (Fig. 2D; Fig. S3). Through this tetramerization event, the DNA-binding domain structure is presumably altered, or zinc increases the affinity of CopY for DNA upstream from the *cop* operon by roughly an order of magnitude (Fig. 1B and 3). These data support the finding that zinc limitation leads to *cop* operon upregulation, as under this zinc limitation, CopY would bind copper more easily, leading to release of the *cop* operon (26).

**FIG 7:**
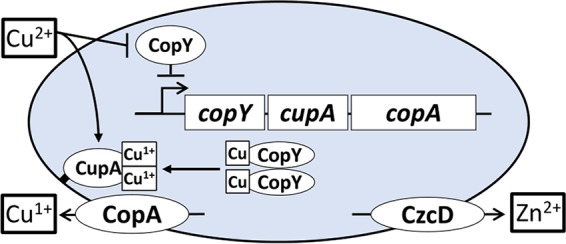
Model of copper efflux involving zinc and CupA. Cu^2+^ enters bacteria and derepresses the *cop* operon. CupA traffics to the membrane and, either en route or once bound to the membrane, chelates copper from CopY or the cytosol. The copper export protein CopA and the zinc export protein CzcD are also made to export Cu^1+^ and Zn, respectively. CupA, if necessary, facilitates the reduction of Cu^2+^ to Cu^1+^ and transfers Cu^1+^ to membrane-bound CopA for export. Once free copper is chelated or exported, apo dimeric or zinc-bound tetrameric CopY can return to repress the *cop* operon.

When Cu^2+^ enters the cell, it might displace bound zinc from CopY to upregulate *copY* operon expression (Fig. 7). This displacement of zinc could also explain the upregulation of the zinc exporter under copper stress (8). However, it is also possible that copper indirectly induces the upregulation of this zinc export system through binding of the MerR family transcriptional regulator *soxR* (*SP_1856*) directly downstream of zinc exporter *czcD* (*SP_1857*) on the negative strand. Like *czcD*, *soxR* is also upregulated under copper stress (8). This copper-specific regulation does not occur through SczA (*SP_1858*), a protein known to regulate *czcD* expression, or CopY, as the promoter sequence is not present (35).

Accordingly, zinc stress, caused either by its addition with copper or by elimination of its efflux system, was detrimental to the pneumococcus under copper stress (Fig. 4). During infection, host zinc and copper levels increase and host deficiencies in these metals lead to an immunocompromised state (36, 37). Silencing of *ATP7A* expression in macrophages decreased their ability to kill *E. coli*, and while limited data exist on the role of phagolysosomal zinc importers in the SLC30 and SLC39 family members, *SLC30A1* expression in macrophages is increased during *M. tuberculosis* infection (10, 38). Zinc’s ability to further repress the *cop* operon gives a glimpse of how the “brass dagger” of copper and zinc toxicity inside the macrophage can be so detrimental to bacteria (Fig. 3) (39, 40). Understanding how zinc and copper interact with CopY to modulate both its structure and its ability to bind DNA will be the subject of future studies.

**FIG 8:**
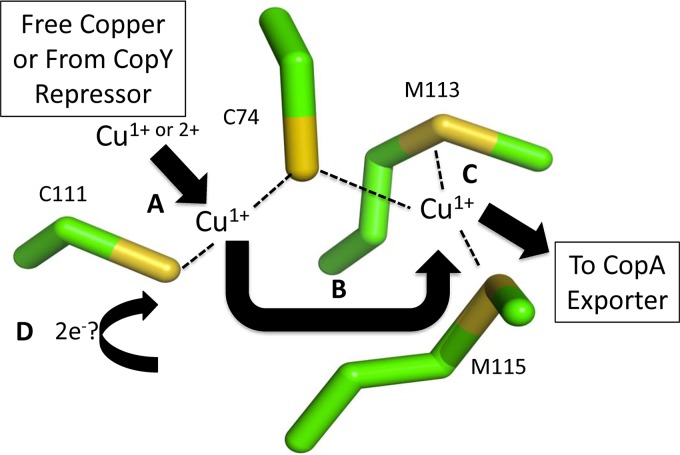
Cupredoxin mechanism of CupA. (A) Chelated copper obtained from CopY or other proteins is reduced to Cu^1+^ by C74 and C111 or oxidizes the donor cysteines. Reducing activity is more efficient with both cysteines present. (B) Cu^1+^ is transferred from site 1 (C74 and C111) to site 2 (C74, M113, and M115) (C) Site 2 holds Cu^1+^ until transfer to CopA for efflux can occur. (D) The oxidized cysteine residues are reduced from a currently unknown source, thus resetting CupA to receive, reduce, and chaperone copper to the CopA exporter. Carbon atoms are green, and sulfur atoms are yellow. PDB code 4F2E.

In the assays used in this study and some other assays studying copper toxicity in the pneumococcus, the copper ion used is Cu^2+^ (3, 8, 13). This form has previously been shown to be toxic in *S. pneumoniae* by mismetallating ribonucleotide reductase, NrdF, an essential protein in aerobic nucleotide synthesis (8). However, the form of copper exported by the bacteria is Cu^1+^, thus creating a constant need for electrons to maintain the reducing environment under copper stress (25). This study showed that CupA can facilitate the reduction of Cu^2+^ to Cu^1+^, thus offering another function for membrane-bound CupA, which acts to chelate copper as it enters the bacteria, and then pass copper to the metal-binding domain of CopA (Fig. 5, 7, and 8) (23). This is supported by previous data that show that the Δ*copY* mutant, in which *cupA* expression is no longer repressed, has significantly higher levels of protein oxidation under increasing copper stress than does wild-type *S. pneumoniae* TIGR4, while the Δ*cupA* mutant has significantly lower levels than wild-type *S. pneumoniae* TIGR4 (8). One hypothesis that is consistent with our findings is that CupA must acquire electrons to reduce the copper that it has taken up as Cu^2+^ for export. Therefore, CupA oxidizes other proteins in the cell to acquire sufficient reducing equivalents, which might explain why the measured copper oxidation level under copper stress is dependent on internal CupA levels. One protein source of these electrons could be thioredoxin (*SP_1776*), which is also upregulated under copper stress (8). However, the identity of the external source of electrons, and any additional protein(s) responsible for reducing Cu^2+^ to Cu^1+^, remains to be established.

CupA has a conserved cupredoxin fold, and there are multiple sources showing that Cu^2+^ can be reduced depending on its binding coordination at sulfur-containing sites, i.e., cysteine- and methionine-rich sequences (41–43). Although there is no established copper import system, there are data supporting the idea that Cu^2+^ enters bacteria through iron siderophores (33, 34). Therefore, given nonreducing conditions where Cu^2+^ is significantly more soluble than Cu^1+^, reduction of Cu^2+^ and efflux of Cu^1+^ could serve to decrease the extracellular pool of Cu^2+^. The result would be relatively insoluble Cu^1+^, which would have more difficulty reentering *S. pneumoniae* than Cu^2+^. This soluble copper accessibility could be one of the reasons why the oxidizing environment of the phagolysosome plays such an important role in the maintenance of toxic metal stress on bacteria.

Our copper reduction assay was dependent on the ability of CupA to reduce and release Cu^1+^ for BCS chelation. In our proposed copper reduction model of CupA, Cu^2+^ is chelated by CupA and reduced to Cu^1+^ (Fig. 8). This process does not negate CupA’s ability to bind Cu^1+^. The reduction process is facilitated by C111 and C74, as shown by the inability of the C74A C111A protein variant to either facilitate reduction of Cu^2+^ or restore CopY binding to DNA in the presence of Cu^2+^ (Fig. 5A to D). CupA’s crystal structure denotes C74 and C111 as high-affinity site 1 for the binding of Cu^1+^ and C74, M113, and M115 as low-affinity site 2 for the binding of Cu^1+^ (23). Our data suggest that the copper-reducing event occurs in conjunction with site 1 and then the resultant Cu^1+^ atom is passed to site 2 to facilitate export via CopA (Fig. 8).

Within the confines of our assay, mutation of M113 at site 2 increased the *V*_max_ and decreased the *K*_m_ of CupA copper reduction relative to those of wild-type CupA. This result was in support of previous studies showing that site 2 is the recipient of the reduced copper, which hands it off to CopA (23). Furthermore, our data suggested, via M113A’s shorter *T*_50_ (the time it takes for 50% of the copper reduction and release to take place at a given copper concentration) and lower *K*_*m*_ than the wild type, that copper reduction by site 1 is enhanced by the ability of site 2 to flux copper. Mutations of site 2 resulted in a reduction in growth in the presence of copper, as previously reported (44). Thus, even though the M113A variant facilitated copper reduction and release faster than the wild-type protein *in vitro*, *in vivo*, M113A CupA likely lost bound copper before it was able to hand copper off to the exporter or was unable to hand off copper to the exporter, leading to decreased bacterial copper resistance.

Mutations of site 1 in our growth and zone-of-inhibition assays also resulted in copper-stunted growth. This difference from previously published results could be due to the concentration (1 M) used in the zone-of-inhibition assay, the medium used, or the mutated residue. Furthermore, the copper-reducing function of CupA explains why, in a Δ*cupA* mutant background of *S. pneumoniae* expressing the *Bacillus subtilis* CopZ metallochaperone, growth cannot be restored in the presence of copper stress (44).

Previous models reported that copper chaperones, such as CopZ in *E. hirae*, carry copper to the repressor for activation of the operon (4, 22). We observed that, at least *in vitro*, the pneumococcal copper chaperone CupA can chelate copper from CopY (Fig. 6A and B). Unlike *E. hirae* CopZ, however, CupA of *S. pneumoniae* has a membrane leader sequence. Full deletions of the CupA membrane leader sequence increase pneumococcal susceptibility to copper stress (23). The membrane leader sequence might facilitate CupA’s interaction with CopA, although this is speculative at this stage. Nevertheless, our current model suggests that in *S. pneumoniae* when CopY binds copper, it does not bind DNA and instead acts as a source of copper for CupA (Fig. 7). CupA reduces Cu^2+^ to Cu^1+^ and transfers copper to CopA for export from the cell as Cu^1+^. After releasing copper to CupA, CopY either binds to the DNA or picks up excess cytosolic Cu^1+^ or Cu^2+^ (Fig. 7). Finally, after the bacteria have sufficiently detoxified copper to a tolerable level, zinc interacts with CopY, allowing it to repress *cop* operon expression (Fig. 7) (36, 45).

## MATERIALS AND METHODS

### Protein homology and domain prediction.

Protein sequences were entered into the NIH Protein Basic Local Alignment Search Tool (BLAST) (16). The BLAST output predicted protein domains and aligned them with similar sequences. Models were generated by using I-TASSER, and models and empirically determined structures were aligned by using PyMOL (18, 46, 47).

### Protein production.

DNA encoding full-length CopY and residues 23 to 123 of CupA was amplified from *S. pneumoniae* TIGR4 DNA and inserted into a pMCSG7 ligation-independent cloning vector (Table S1). The first 22 amino acids are a leader sequence that makes the protein insoluble. Site-directed mutagenesis was performed to produce C74A, C111A, M113A, M115A, and C74A C111A mutations in the gene encoding CupA. Vectors for CopY and CupA production were used to transform *E. coli* BL21 Gold DE3 cells (Stratagene) with selection on ampicillin plates (100 mg/ml). A single colony was used to inoculate a 100-ml flask of Luria broth (LB) containing ampicillin (100 mg/ml), and the cells in the culture were grown overnight. Cell were collected by centrifugation at 3,000 × *g*, and the supernatant was discarded. The resultant pellet of cells was used to inoculate a 2.8-liter shaker flask of LB containing ampicillin (100 mg/ml). Cells were grown in Terrific broth at 37°C until the optical density at 600nm (OD_600_) of the culture was 0.4. The temperature was lowered to 17°C for overnight growth, and protein production was induced by adding 0.3 mM isopropyl-β-d-thiogalactopyranoside. After overnight growth, cells were harvested by centrifugation at 6,000 × *g* for 20 min at 4°C. The supernatant was discarded, and cell pellets were stored at −80°C.

Cell pellets were thawed by using a buffer consisting of phosphate-buffered saline (PBS) containing 10 mM imidazole, 5% glycerol, 200 U of Benzonase nuclease (Novagen), 1 mM phenylmethylsulfonyl fluoride, and protease inhibitor tablets (Roche). Cells were passed twice through a microfluidizer and centrifuged at 16,000 × *g*. The soluble fraction was filtered through a 0.22-μm filter and nickel purified on gravity HisPur Ni-nitrilotriacetic acid (NTA) resin (Thermo Fisher) by using PBS containing 250 mM imidazole and 5% glycerol. Elution fractions were concentrated to 10 ml and dialyzed into either 1 liter of PBS containing 5% glycerol or 1 liter of 50 mM Tris (pH 7.5), 100 mM NaCl, and 5% glycerol each at least three times. The results of an SDS-PAGE assay confirmed that the protein was 95% pure, and gel filtration assay results confirmed that the protein was not a soluble aggregate. Purified proteins were concentrated to ~500 to 1,000 µM for CupA and the corresponding variants and ~150 µM for CopY and stored at −80°C.

As needed, eluted His-tagged CupA was incubated with tobacco etch virus (TEV) protease at 1 mg/ml to cleave the 6×His tag and dialyzed in 1 liter of 50 mM Tris-HCl (pH 7.5), 100 mM NaCl, and 1 mM dithiothreitol (DTT) for 18 h at 4°C. The sample was then dialyzed four times in 50 mM Tris-HCl (pH 7.5) and 100 mM NaCl to eliminate the DTT. Samples were then filtered and passed through a nickel column preequilibrated with 50 mM Tris-HCl (pH 7.5) and 100 mM NaCl. The flowthrough as CupA was collected and concentrated to ~500 µM and frozen at −80°C. When necessary, samples and the corresponding buffers were treated with a gravity flow column consisting of Chelex 100 resin.

### Induction of *copA*.

*S. pneumoniae* in THYB was incubated with copper, silver, and zinc at 100 µM for 30 min. Bacteria were collected, and RNA extraction and quantitative real-time PCR were performed as previously described (3). SuperScript III First-Strand Synthesis SuperMix (Invitrogen) was used to synthesize cDNA from the isolated bacterial RNA (50 ng/pl). SYBR green (Invitrogen) was used to monitor DNA amplification on an ABI Prism 7300 real-time PCR machine (Applied Biosystems). After samples were normalized relative to *gyrA* expression, fold change was obtained by the ΔΔ*C*_*T*_ method.

### CD.

CopY in PBS with 5% glycerol was chelated and diluted to 10 µM with or without 50 µM CuSO_4_, ZnSO_4_, and AgCl_2_. A wavelength scan from 200 to 250 nm was performed with a 62 DS CD spectrometer (Aviv) at 23°C with a 15-s averaging time. The equation used to calculate mean residue ellipticity was θ × MW/10 × *c* × *l* × *r*, where θ is the experimental wavelength, MW is the molecular weight (18,000), *c* is the concentration (10 µM or 0.18 mg/ml), *l* is the path length (1 mm), and *r* is the number of residues (133 from CopY and 24 from the His tag and TRV protease cleavage site).

### EMSA.

DNA leading strands *pSP_0727* (TCTATAATTGACAAATGTAGATTTTAAGAGTATACTGATGAGTGTAATTGACAAATG/3Bio/) and *pSP_1541* (GCGAACCTCACTTACCCCTTGCAAAGTCTTGGGGTCATTAGA/3Bio) were purchased from IDT and annealed to the lagging strand partner CATTTGTCAATTACACTCATCAGTATACTCTTAAAATCTACATTTGTCAATTATAGA (SP0727) or TCTAATGACCCCAAGACTTTGCAAGGGGTAAGTGAGGTTCGC (SP_1541) by heating the reaction mixture containing the strands to 95°C and then reducing the temperature by 1°C/min to 22°C. DNA was then diluted to the desired concentration. Next, 0.5× Tris-borate-EDTA (TBE) electrophoresis buffer with no EDTA (so as not to chelate the metals used in the assay) was prepared and used as the buffer for both the 6% TBE DNA retardation gel (Invitrogen) and transfer to Zeta Probe blotting membrane (Bio-Rad). Unless indicated otherwise in the figure legend, 50 µM CopY was added with 50 ng of promoter DNA and brought to the desired volume of 20 µl with Tris-buffered saline (TBS), taking into account future changes in volume by metals added (from a 1 mM stock) and/or CupA (100 µM), if applicable. Samples were incubated at 4°C for 5 min at each step, loaded onto a TBE polyacrylamide gel, and then electrophoresed at 40 V for 120 min (100 V for 60 min for zinc). The polyacrylamide gel was incubated in distilled H_2_O (dH_2_O) with 0.02% ethidium bromide (AMRESCO) for 10 min and washed twice for 5 min each time in dH_2_O, and the gel was visualized via UV on a Gel Doc XR+ system (Bio-Rad).

### SEC-MALS.

SEC-MALS experiments were performed with a WTC-010S5 (molecular mass range, 100 to 100,000 Da) size exclusion column (Wyatt Technologies, Santa Barbara, CA, USA) with three detectors connected in series, i.e., an Agilent 1200 UV detector (Agilent Technologies, Santa Clara, CA), a Wyatt DAWN-HELEOS MALS detector, and a Wyatt Optilab rEX differential refractive index detector (Wyatt Technologies, Santa Barbara, CA, USA). The DAWN-HELEOS MALS detector uses a laser wavelength of 658 nm and was calibrated using the manufacturer's specifications. The columns were equilibrated with 50 mM Tris-HCl (pH 7.5), 100 mM NaCl, and 5% glycerol with or without 100 µM zinc, and the experiment was conducted at 25°C. A 100-µl sample was placed on the column with the autosampler, and a flow rate of 0.40 ml/min was maintained throughout the experiments. Protein in the eluent was detected and measured via light scattering, absorbance at 280 nm, and refractive index determination, and the data were recorded and analyzed by using Wyatt Astra software (version 6.0.5.3). The refractive index increment, *dn*/*dc*, was taken as 0.185 ml/g to measure the concentration of the protein sample. EASI Graphs (Astra software) were exported and plotted as a molar mass distribution superimposed on (i) a chromatogram of absorbance at 280 nm versus the elution volume and (ii) the same graphs with the addition of the light scattering and refractive index signal distributions (48).

### Fluorescence polarization assay.

DNA was annealed as previously described for EMSAs but with a 6-carboxyfluorescein (FAM) tag on the leading strand. Samples were diluted in Chelex 100-treated buffer containing 50 mM Tris-HCl (pH 7.5), 100 mM NaCl, and 5% glycerol to obtain the desired CopY concentration (25 to 50 or 2 µM). Experiments were run in quadruplicate 20-µl aliquots pipetted into a 384-well black-bottom plate (Corning), and results were read on a PHERAstar FS plate reader with the excitation wavelength set at 495 nm and the emission wavelength set at 520 nm. Results were normalized to the maximal DNA binding obtained by the one-site binding hyperbola equation or sigmoidal equation.

### Bacterial constructs.

The *S. pneumoniae* TIGR4 Δ*czcD* mutation was created via the splicing by overhang extension (SOE) PCR method (Table S1). Fragments approximately 1 kb upstream and downstream of the target gene were amplified and spliced to an erythromycin resistance cassette. SOE PCR products were subsequently used to transform *S. pneumoniae* TIGR4. Briefly, for transformation, bacteria were grown to an OD_600_ of 0.1 and induced with competence-stimulating peptide 2 (CSP2) for 14 min. The SOE PCR product was then added to bacteria for a 2-h recovery period at 37°C before plating of bacteria on tryptic soy agar (TSA) blood agar plates with 20 µg/ml neomycin and 1 µg/ml erythromycin. Single colonies were verified by PCR to confirm correct insertion of the SOE PCR product and deletion of the target gene (49).

### Growth curves.

Using THYB (30 g of Todd-Hewitt broth [Sigma], 2 g of yeast extract [Sigma], and 1 liter of dH_2_O, pH 6.5), *S. pneumoniae* was grown to an OD_620_ of 0.1 and diluted 1:50 into THYB with or without metal stress. The OD_600_ was measured after 18 h or every 30 min for 10 h of growth in a 96-well plate on a BioTek Cytation 5 and normalized to maximal growth without metal stress. Data are representative of four individual experiments.

### Cupredoxin assay.

For *V*_max_ and *K*_*m*_ studies, BCS was added at 1 mM with 50 µM CupA (or variants thereof) and various amounts of CuSO_4_ (5 to 625 µM) in TBS to a final volume of 1 ml to a cuvette and the absorbance at 490 nm was read in a spectrophotometer with TBS, CuSO_4_, and BCS used as the blank. For maximal binding (*B*_max_) and *T*_50_ in the timed measurements, TBS, 1 mM BCS, 50 CupA (or variants thereof), and 50 µM CuSO_4_ (in that order) were added to a cuvette in a final volume of 1 ml and monitored over time, with TBS with CuSO_4_ and BCS used as the blank. Data were plotted as the average of three individual experiments.

### CupA mutants.

The Δ*cupA* mutants in the *S. pneumoniae* TIGR4 background were made as described in reference 3 (Table S1). Full-length *cupA* was cloned from *S. pneumoniae* TIGR4 and ligated into pABG5 by using EcoRI. Site-directed mutagenesis was performed as previously described to obtain mutants. After incubation with CSP2 as described above, the pABG5 vector containing sequence-confirmed mutant forms was added to competent cells of the *S. pneumoniae* Δ*cupA* mutant grown to an OD of 0.1. After a 2-h recovery, bacteria were plated on TSA blood agar plates containing kanamycin at 400 µg/ml.

### Zone-of-inhibition assay.

Bacteria were grown in THYB at pH 6.5 to an OD_620_ of 0.3. A 100-µl volume of culture was then spread onto a blood agar plate. A disc of filter paper with 10 µl of 1 M CuSO_4_ was placed on the plate. After overnight growth, the distance between the outer edge of the disc and the bacterial growth was reported as the zone of inhibition.

### Inductively coupled plasma mass spectrometry.

First, 10 nmol of chelated CopY with the 6×His tag was incubated with or without 50 nmol of CuSO_4_ for 10 min at 4°C, and then the CopY-Cu mixture or apo chelated CopY was incubated with 100 µl of Ni-NTA Superflow resin for 10 min. Samples were gently centrifuged at 500 × *g* for 1 min and washed three times with a 10× resin volume in 50 mM Tris-HCl (pH 7.5), 100 mM NaCl, and 5% glycerol. Chelated, TRV protease-cleaved CupA (no 6×His tag) was incubated with both the CopY-Cu mixture or apo chelated CopY bound to Ni-NTA resin for 10 min at 4°C. Samples were gently centrifuged at 500 × *g* for 1 min, and CupA was collected. CupA samples and eluted CopY samples were then mixed in 5 ml of 1% nitric acid and filtered for spectrometry (820 ICPMS System; Varian, Inc.). The fold difference between the amounts of copper bound to CupA in the non-copper-treated and copper-treated CopY samples was determined. Data shown represent the average of three individual experiments.
