# Supplementary figures and images for: Copper Chaperone CupA and Zinc Control CopY Regulation of the Pneumococcal cop Operon
Source: mSphere. 2017 Oct 18;2(5):e00372-17. doi: 10.1128/mSphere.00372-17 (PMC5646241; doi:10.1128/mSphere.00372-17)

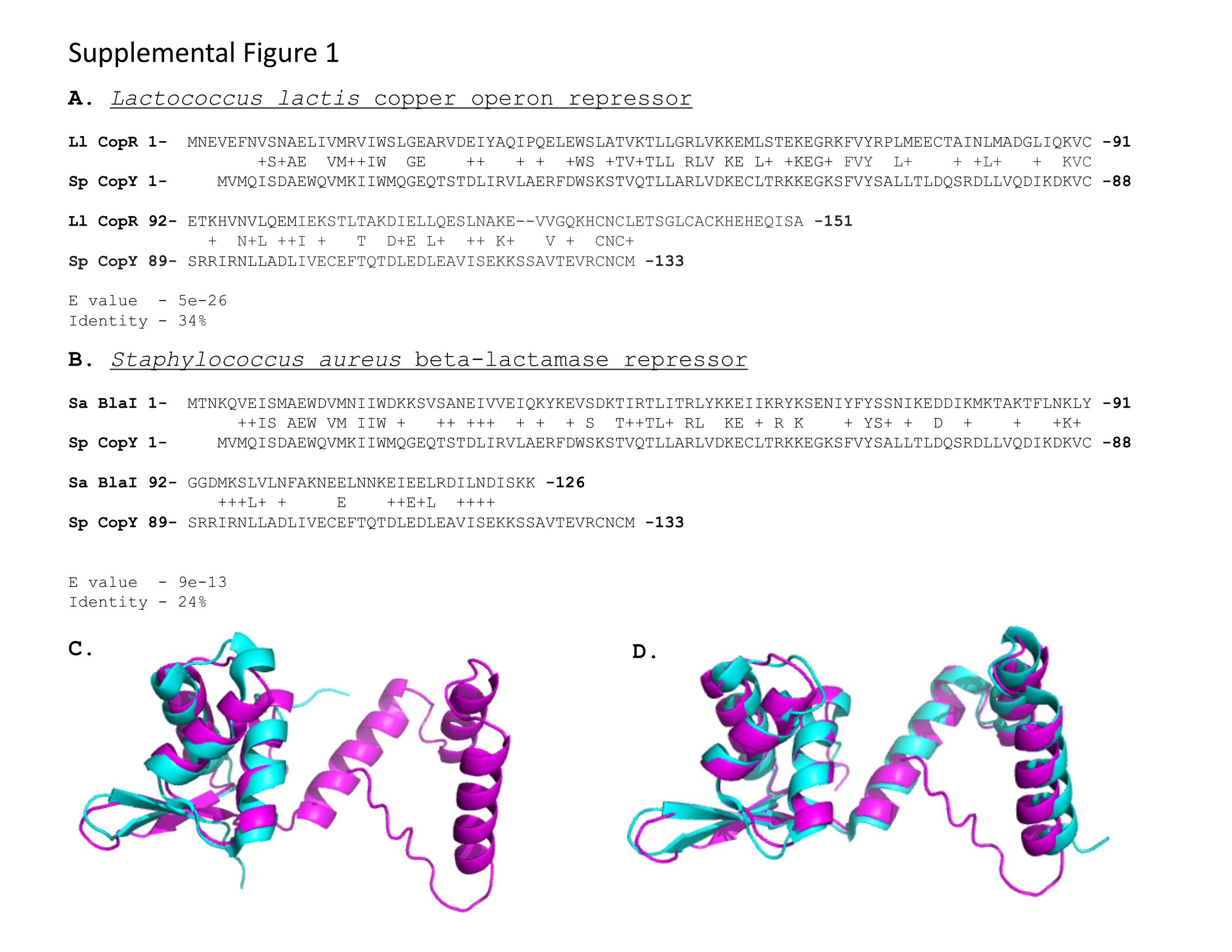

Supplement: FIG S1 [file sph005172384sf1.tif]

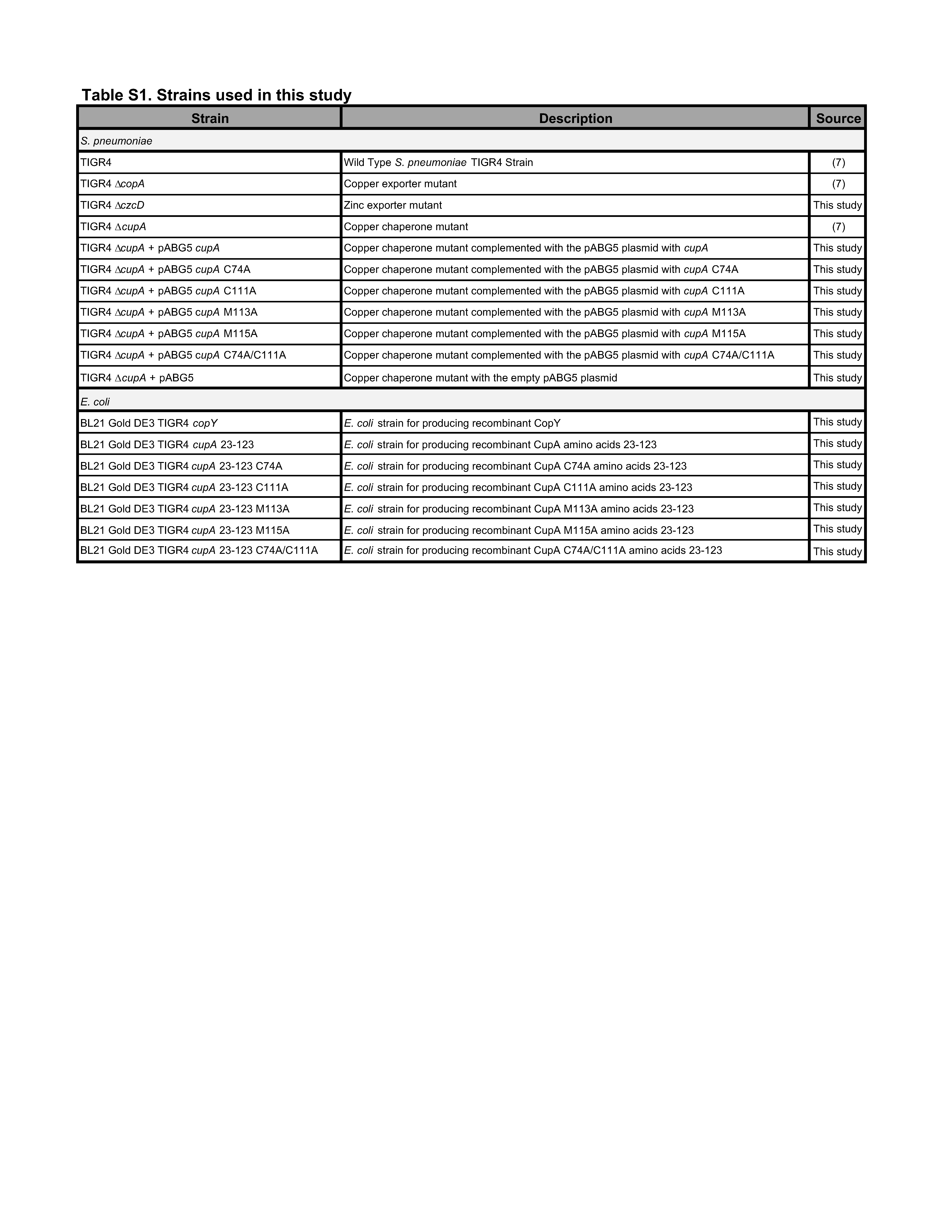

Supplement: TABLE S1 [file sph005172384st5.tif]

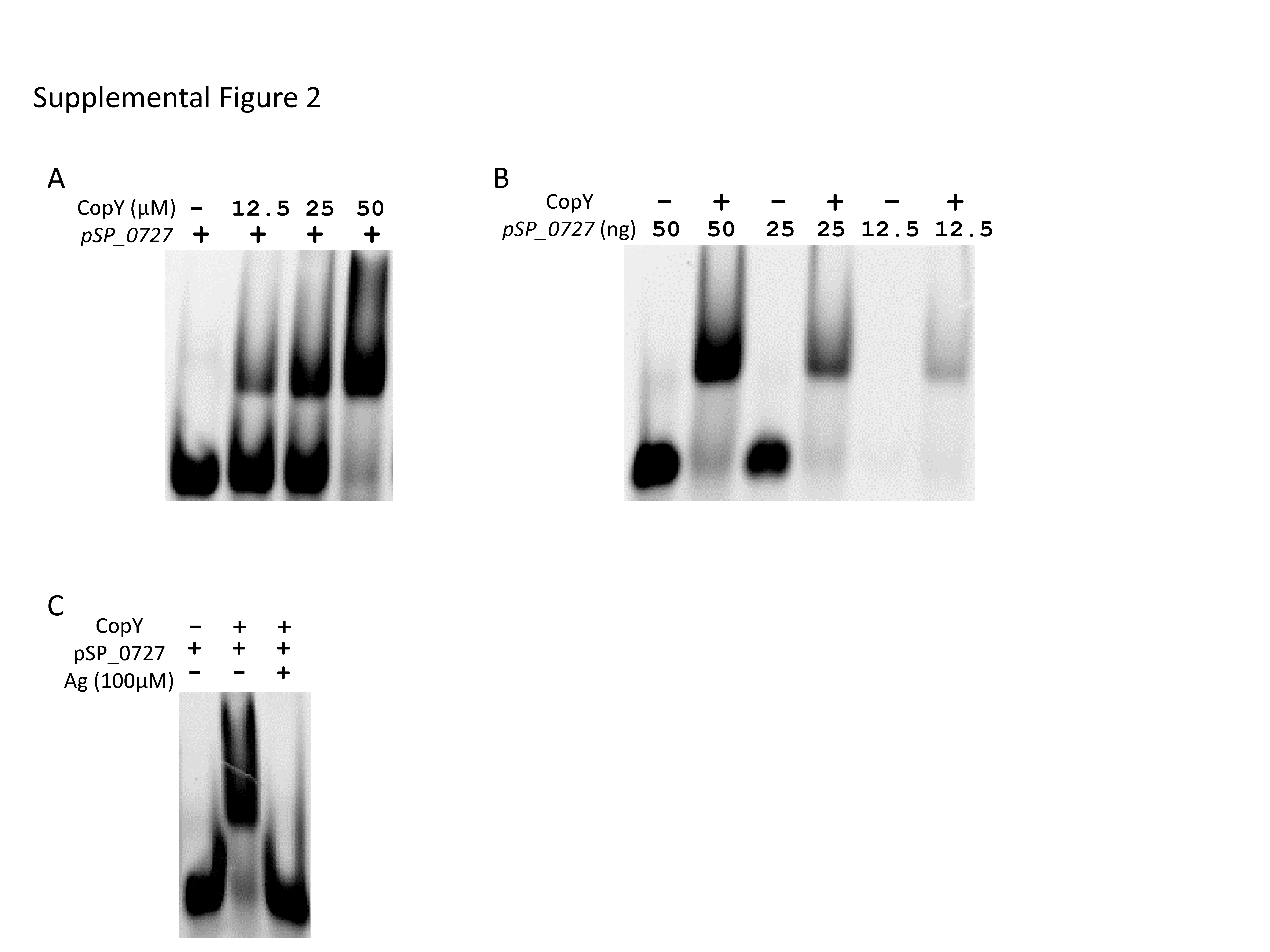

Supplement: FIG S2 [file sph005172384sf2.tif]

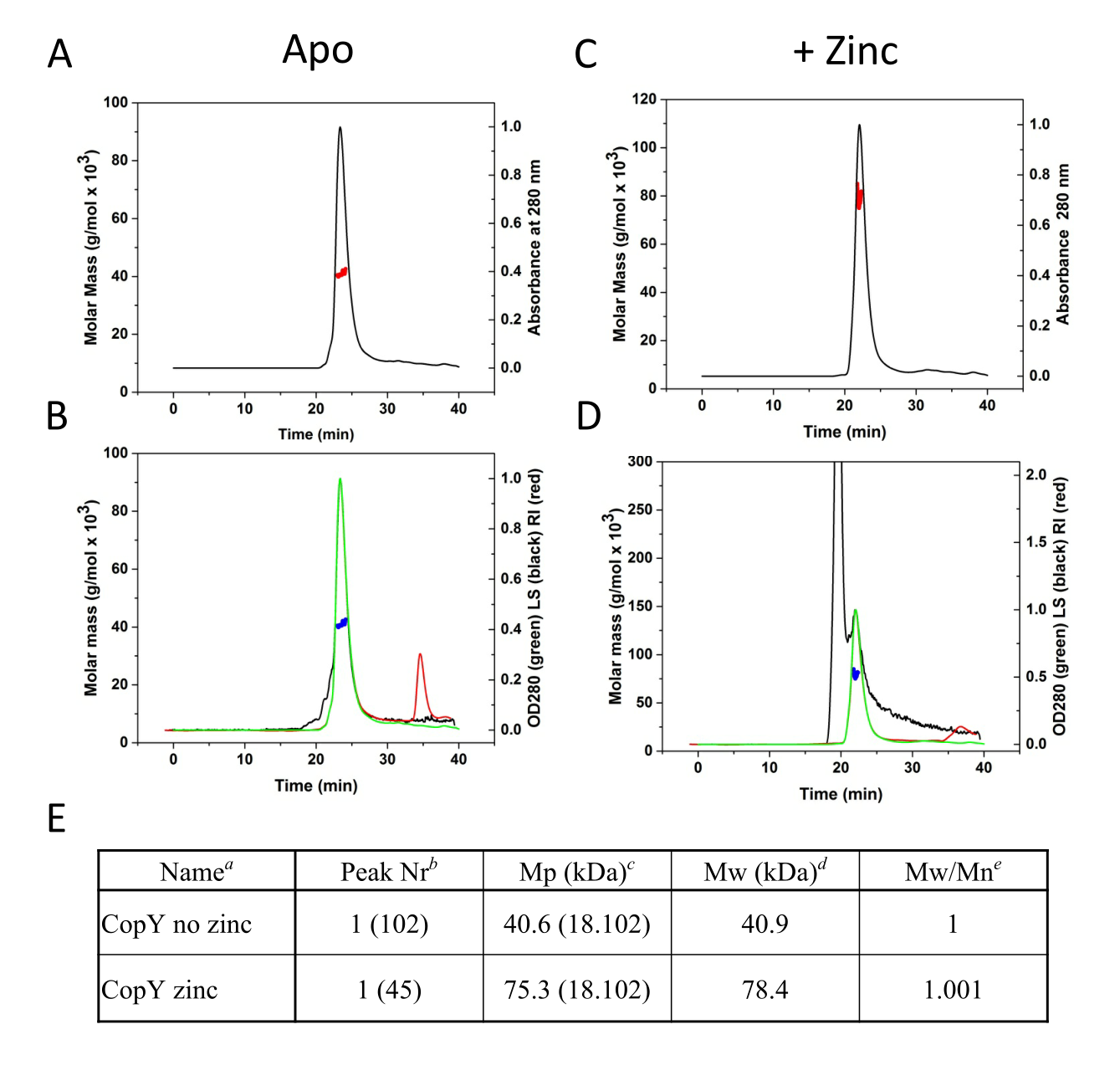

Supplement: FIG S3 [file sph005172384sf3.tif]
